# Structure-Based Drug Design Targeting Topoisomerase II Alpha: Discovery of Potential Antitumor Xanthone Derivatives

**DOI:** 10.3390/molecules31101670

**Published:** 2026-05-15

**Authors:** Thi Thuy Huong Le, Thi Nguyet Hang Nguyen, Minh Quan Pham, Thi Thu Thuy Tran, Tu Thi Dinh, Thi Hoai Van Tran, Van Lang Tran, Quoc Long Pham

**Affiliations:** 1 Institute of Chemistry, Vietnam Academy of Sciences and Technology, 18 Hoang Quoc Viet, Nghia Do, Hanoi 100000, Vietnam; thuyhuong0102sp2@gmail.com (T.T.H.L.); pham-minh.quan@ich.vast.vn (M.Q.P.); thuy.tran@ich.vast.vn (T.T.T.T.); dinhtu0309@gmail.com (T.T.D.); 2 Faculty of Chemistry, Graduate University of Science and Technology, Vietnam Academy of Sciences and Technology, 18 Hoang Quoc Viet, Nghia Do, Hanoi 100000, Vietnam; 3 Faculty of Pharmaceutical Chemistry and Technology, Hanoi University of Pharmacy, 13-15 Le Thanh Tong, Cua Nam, Hanoi 100000, Vietnam; hangntn@hup.edu.vn; 4 Faculty of Basic Sciences, Vietnam University of Traditional Medicine, Ministry of Health, Tran Phu, Dai Mo, Hanoi 100000, Vietnam; hoaivantt@ms.vutm.edu.vn; 5 Ho Chi Minh City University of Foreign Languages—Information Technology, Ho Chi Minh City 700000, Vietnam; langtv@huflit.edu.vn; 6 Laboratory of Biophysics, Institute for Advanced Study in Technology, Ton Duc Thang University, Ho Chi Minh City 700000, Vietnam; 7 Faculty of Pharmacy, Ton Duc Thang University, Ho Chi Minh City 700000, Vietnam

**Keywords:** anticancer, xanthone compounds, phenolic compounds, topoisomerase IIα, computer-aided drug design, pharmacophore modeling

## Abstract

Cancer represents a major global health challenge, contributing to an estimated 19 million new cases annually. While conventional chemotherapeutic approaches continue to advance, target-based therapeutic strategies are increasingly recognized as effective pathways in modern drug development. A prominent biological target in current anticancer research is the selective inhibition of Topoisomerase II alpha (TOP2A). TOP2A, a crucial DNA topoisomerase, is vital for maintaining genomic integrity by mediating the cleavage and re-ligation of double-stranded DNA during essential cellular processes, such as DNA replication and transcription. Inhibiting TOP2A effectively disrupts these processes, leading to cell death. This study employed computer-aided drug design approaches to virtually screen a library of 3000 xanthone derivatives against the TOP2A target, and the results were preliminarily validated through cytotoxicity assays on the A549 and HepG2 cancer cell lines. The computational methods utilized included molecular docking, pharmacological modeling, molecular dynamics simulations, and steered molecular dynamics simulations. The virtual screening identified two highly promising HIT compounds, CID162372098 and CID156619937, that exhibited the most favorable interactions and stability profiles in relation to the TOP2A active site. The experimental results demonstrated that both hit compounds effectively exhibited significant anti-proliferative activities against the HepG2 cell line, with IC50 values of 9.54 ± 0.26 µg mL^−1^ (CID162372098) and 10.03 ± 0.36 12.69 ± 0.31 µg mL^−1^ (CID156619937), respectively. Collectively, these findings demonstrate the potential of xanthone-based scaffolds as inhibitors of TOP2A and provide a rational framework for the screening and development of novel anticancer agents.

## 1. Introduction

With the development of social life, health-related concerns have become increasingly prominent, particularly in light of the rising incidence of various cancers in recent years and the growing number of reported cases. Cancer remains one of the most life-threatening diseases of the 21st century, and the development of effective therapeutic strategies continues to pose a significant challenge. According to data on the number of new cancer cases in 2022 from The Global Cancer Observatory (GLOBOCAN), there are nearly 20 million new cases and 9.9 million cancer deaths worldwide, of which Asia is still the flashpoint for cancer, accounting for almost 50% of all new cases. In 2022, Vietnam recorded 180,480 new cases with 120,184 deaths from cancer. The rate of breast cancer, being the most common, accounted for 25.8% of cases in women. This statistic makes Vietnam one of the countries with high cancer rates globally, putting inordinate pressure on the health system and socio-economy. Not only in Vietnam, but cancer is also a significant concern in most low- and middle-income countries, accounting for about two-thirds of the total number of new cases globally [1]. It might be found that the cause of this problem is limited access to advanced treatment methods, especially the affordable and accessible anticancer drugs.

In eukaryotic cells, DNA topoisomerases are crucial enzymes that regulate DNA supercoiling during replication, transcription, and chromosome segregation [2,3]. In a cell, topoisomerase I (TOP1) cuts a single DNA strand without energy consumption, and topoisomerase II (TOP2) cleaves both DNA strands, requiring ATP hydrolysis and Mg^2+^ as a cofactor [4]. Human cells express two TOP2 paralogs, alpha and beta, with Topoisomerase IIα (TOP2A) being particularly relevant in cancer due to its role in cell proliferation and chromosome segregation [4,5]. TOP2A is a homodimer with three important gates: the N-gate containing the ATP-binding and hydrolysis site. The DNA-gate contains the TOPRIM domain (residues 455–572) corresponding with the Mg^2+^ binding sites, and the TOP2A catalytic domain (residues 715–1171) with important evolutionarily conserved residues L794, D797, A801, and R804, Y805, and I856 for catalysis and complex intermediate stabilization. C-gate is involved in DNA phosphorylation and geometry recognition [4,6,7]. The catalytic mechanism of TOP2A involves a temporary double-strand DNA break to allow DNA to unwind, followed by resealing [8,9]. This process requires Mg^2+^ and ATP as cofactors [6]. The intermediate transient state of the enzyme-DNA cleavage complex is usually short-lived and immediately becomes reversible. It is the covalent bond of enzyme-DNA that plays a crucial role in the TOP2A mechanism. It preserves the bonding energy of the deoxyribose-phosphate DNA backbone and prevents recombination and translocation mutations due to DNA strand cleavage from the enzyme, leading to the preservation of the integrity of the genetic material during the cleavage process [8]. However, the balance of the DNA-TOP2A complex is crucial in the cell, with too little of the complex leading to mitotic failure, while excessive accumulation causes conversion to permanent DNA breaks, chromosomal translocations, and apoptosis [6,10]. Therefore, TOP2A is not only important for cell survival but also a key therapeutic target in oncology, where perturbation of its cleavage–religation cycle underlies both its anticancer utility and the risk of drug-induced genotoxicity.

Xanthone, a naturally occurring heterocyclic compound with the chemical formula C_13_H_8_O_2_. This compound features a symmetrical skeleton structure based on the 9H-xanthen frame. To date, around 650 natural sources of xanthone have been identified [11], and they exhibit a broad range of pharmacological effects due to their varied functional groups, substituent positions, oxygenation, and ability to bind to different receptors. Consequently, research on both natural and synthetic xanthone derivatives primarily aims to uncover new drug candidates with anti-inflammatory, antioxidant, and notably, anticancer properties [11,12]. A prime example of their potent anticancer activity is gambogic acid (GA), a prominent xanthone from *Garcinia hanburyi*, which has been used in traditional medicine and shown promising anticancer results in clinical trials, targeting pathways like autophagy and angiogenesis. Despite GA’s potent activity, its low water solubility is a current focus for structural optimization to improve its efficacy [13]. Therefore, the xanthone scaffold continues to represent a valuable and promising structural framework for anticancer drug discovery and development.

Advances in Computer-Aided Drug Design (CADD) have driven a significant transformation in the pharmaceutical industry, particularly with the advent of modern digital technologies and the rapid expansion of bioinformatics applications worldwide since the mid-20th century [14]. CADD appears to address many challenges of traditional drug discovery, which is characterized by exorbitant costs (approximately one billion dollars per drug), long timelines (typically 8–12 years for clinical drugs), and high clinical trial failure rates [15,16]. The utilization of supercomputers, parallel processing, sophisticated software and algorithms, and other technological advancements in CADD made drug discovery and the development process simpler, which facilitates understanding and simulation of crucial molecular interactions and binding affinities between target proteins and potential ligands. Furthermore, advanced progress in machine learning has greatly enhanced the analysis of the vast pharmaceutical data generated during drug discovery, further amplifying CADD’s capacity to reduce expenses, shorten development cycles, and improve success rates [17]. With the rapid advancement of computer science, the application of computational tools in drug development is expected to become increasingly prevalent in the near future.

## 2. Results

**Figure 1:**
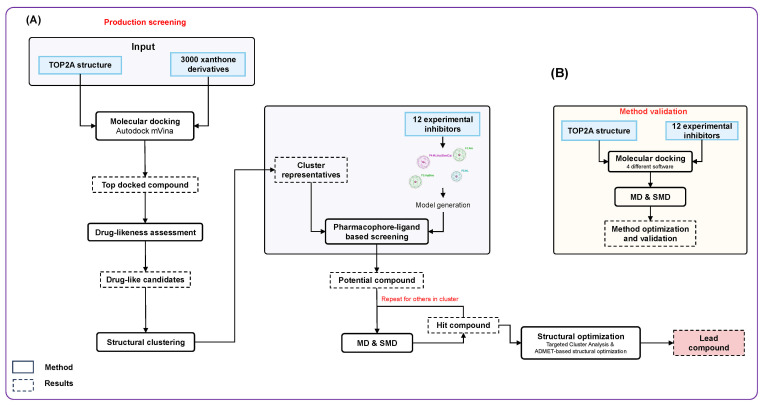
Workflow of predicting a potential inhibitor for TOP2A. (**A**) Production screening scheme was applied to estimate HIT compounds; (**B**) method validation scheme was applied to optimize and validate methods before conducting production screening.

In this study, molecular dynamics simulations and steered molecular dynamics simulations combined with structural analysis methods were used to identify potential inhibitors of TOP2A. An initial method validation study (Figure 1B) on 12 experimental inhibitors was first conducted to optimize and validate the calculated parameters for the molecular docking, molecular dynamics and steered molecular dynamics simulation methods before applying them to the large-scale screening (Figure 1A). In the large-scale screening, the binding free energy calculation, structure-based screening and pharmacokinetics were subsequently validated for the 3000 xanthone data. The predicted inhibitors showed high confidence for experimental studies and demonstrated the molecular mechanism of TOP2A inhibitory interaction.

### 2.1. Method Validation

#### 2.1.1. Molecular Docking Benchmark

To predict the binding of ligand to target protein and to rapidly estimate their binding affinities and binding poses, docking simulations can be applied, referring to the previous studies [18,19]. Initially, a preliminary method validation was performed to evaluate the performance of several docking methods, including a modified version of AutoDock Vina (mVina) [20], Vina [21], Vinardo [22], and AD4 [23], by docking a set of 12 known inhibitors to TOP2A. In particular, the accuracy of each method was assessed by comparing the predicted docking data to available experimental results, which are presented in Table 1. The binding free energy value of each substance is calculated based on the IC_50_ value from the experiment by using the Cheng–Prusoff equation [24].

**Figure 2:**
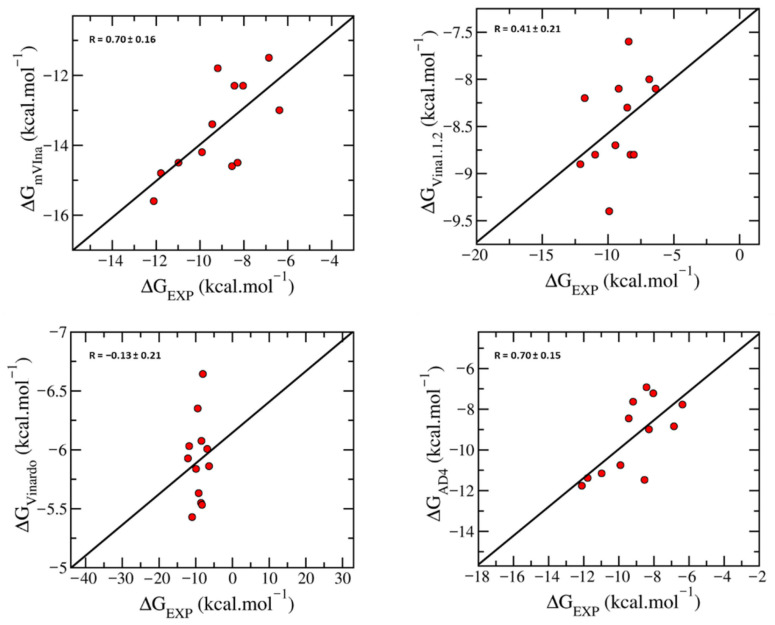
The coefficient between docking and experimental ligand-binding free energy by four molecular docking methods.

As illustrated in Figure 2, mVina demonstrated the highest correlation coefficient (R=0.70) between the calculated docking and experimental binding affinity. Consequently, mVina was selected as the preferred method for docking a library of 3000 xanthone compounds.

The docking poses of the 12 experimental inhibitors with the TOP2A target are shown in Figure S1. The ligands primarily form hydrogen bonds and hydrophobic interactions, which contribute to ligand–protein binding [25]. Key residues involved in binding include nucleotides (DC8, DG13, and DG7), Arg487, Met762, and Met766. These amino acids are located in the domain responsible for interactions with DNA and Mg^2+^ and are closely associated with the catalytic activity of TOP2A.

#### 2.1.2. MD and SMD Validation

Molecular docking provides only a static representation of ligand–protein interactions and does not fully capture system dynamics. Therefore, three independent molecular dynamics (MD) simulations (100 ns each) were performed to better characterize system behavior under physiological conditions [26,27,28].

The simulation trajectories were analyzed using root mean square deviation (RMSD), root mean square fluctuation (RMSF), and radius of gyration (Rg) (Figure S2a–c). The RMSD results indicate that all systems reached a stable equilibrium after approximately 50 ns. The RMSF profiles showed that most residues exhibited low fluctuations, while higher fluctuations were mainly observed in the flexible loop and terminal regions. Importantly, residues in the binding site remained relatively stable across the three independent trajectories. The Rg profiles remained nearly constant throughout the simulations, indicating that the overall compactness of the protein was preserved.

**Figure 3:**
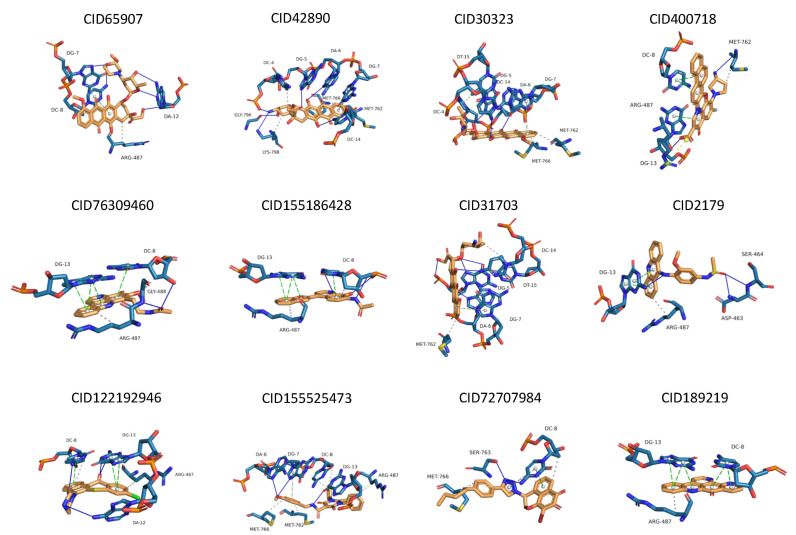
MD refined structure binding pose between the experimental inhibitors on TOP2A. Hydrogen bond–navy line; hydrophobic interaction–black dashed line, π-stracking—green dashed line. DG, DC, DT, and DA are nucleotides in which DA: adenine, DT: thymine, DG: guanine, and DC: cytosine.

Representative structures from each trajectory were selected to analyze binding modes (Figure 3). Minor conformational changes were observed during the simulations; however, key interactions between ligands and the binding site residues were largely preserved. After MD, seven common residues were identified as important for ligand interaction, including Arg487, Met766, Met762, DA6, and nucleotides DG7, DC8, and DG13.

The binding affinity of 12 experimental inhibitors was then estimated using guided steered-MD simulations, specifically a technique known as fast pulling of ligand (FPL) [29,30]. In this study, FPL was used to calculate the binding free energies of 12 known inhibitors to TOP2A. SMD simulation using typical configurations, clustered from the last 50 ns of MD simulation, an external harmonic force was applied over an 800 ps simulation along the *z*-axis, causing the ligand to dissociate from the active site. The pulling force increased rapidly and reached a maximum value around 200ps before quickly returning to zero, indicating the completion of ligand dissociation (see Figure S3). The maximum pulling work (W) and average pulling force (F_max_) for each compound are detailed in Table 2.

**Figure 4:**
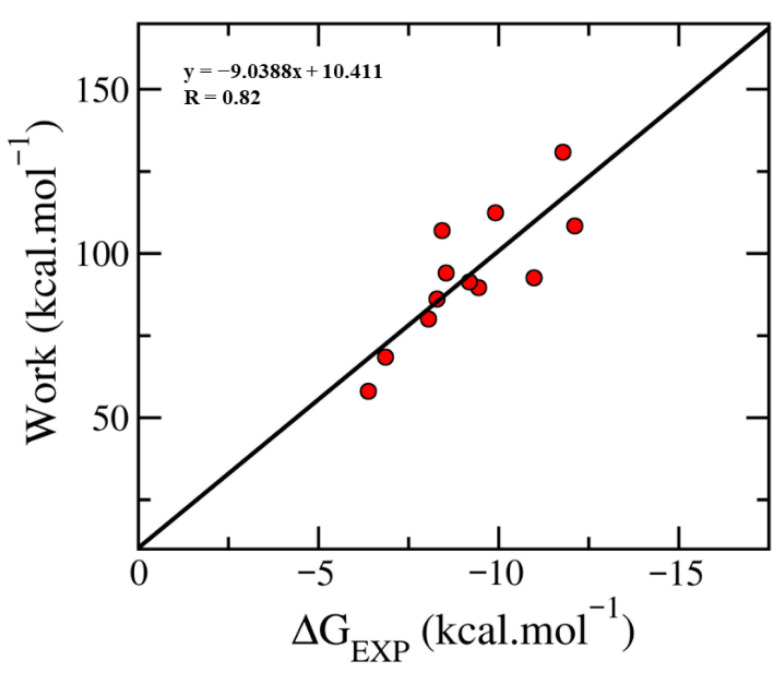
Correlation between pulling work (W) and experimental binding free energy (ΔG_exp_).

A significant linear correlation (R = 0.82 ± 0.13) between the W and experimental binding free energy was calculated (Figure 4), validating FPL as a suitable approach for TOP2A ligand binding affinity estimation. Therefore, a regression model was established to predict ΔG_FPL_ directly from W, with stronger inhibitors requiring higher pulling work to dissociate.

ΔG_FPL_ = −0.1106 × W + 1.1518 (1)

where ΔG_FPL_ is predicted binding free energy (kcal mol^−1^), W is the average of pulling work (kcal mol^−1^).

All parameters of mVina molecular docking, molecular dynamics and steered-molecular dynamics simulation were used as reference standards for the entire large-scale screening, ensuring that the evaluation criteria in the subsequent steps were based on validated data.

### 2.2. Large-Scale Screening and Analysis

#### 2.2.1. Docking and RO5 Filtering

The mVina molecular docking method was utilized to calculate the binding free energy of 3000 xanthone compounds against the TOP2A target. The results were compared with the reference ligand, etoposide, a known TOP2A inhibitor, co-crystallized in the TOP2A complex (PDB ID: 5GWK). According to the ranking criteria of Autodock Vina, the more negative docking energy suggests a higher binding affinity of the compound towards the targeted receptor. A total of 157 compounds exhibited more negative binding free energies than etoposide (ΔG_mVina_ = −15.5 kcal mol^−1^) and were selected. Subsequently, these compounds were filtered using Lipinski’s Rule of Five to predict their potential for oral absorption. Out of the 157 candidates, only 32 compounds satisfy at least three of the four criteria for favorable physicochemical properties (molecular weight < 500 Da, ≤5 hydrogen bond donors, ≤10 hydrogen bond acceptors, and a logP ≤ 5), as detailed in Table S1.

#### 2.2.2. Clustering and Representative Selection

The 32 drug-like xanthone derivatives obtained after RO5 filtration, together with etoposide, were clustered into 18 groups based on fragment fingerprint similarity (≥80%) using a hierarchical clustering algorithm [31]. Representative compounds (medoids) were selected for each cluster (Figure S4), ensuring structural diversity while reducing redundancy for subsequent analysis.

Cluster 3, consisting of five closely related analogs, was identified as a dense region in the SALI network (Figure S5), indicating high structural similarity among its members. These compounds share a common xanthone scaffold with variations in substituents such as carboxyl, ester, halogen (F), and methoxy (OCH_3_) groups.

Structural differences within this cluster arise from modifications such as carboxyl-to-ester conversion, cis–trans isomerization, and positional or functional substitution (e.g., para- to meta-fluorine shift or F → OCH_3_ replacement). Despite these variations, the core framework remains conserved, highlighting the structural coherence of this cluster.

#### 2.2.3. Ligand-Based Pharmacophore Screening

**Figure 5:**
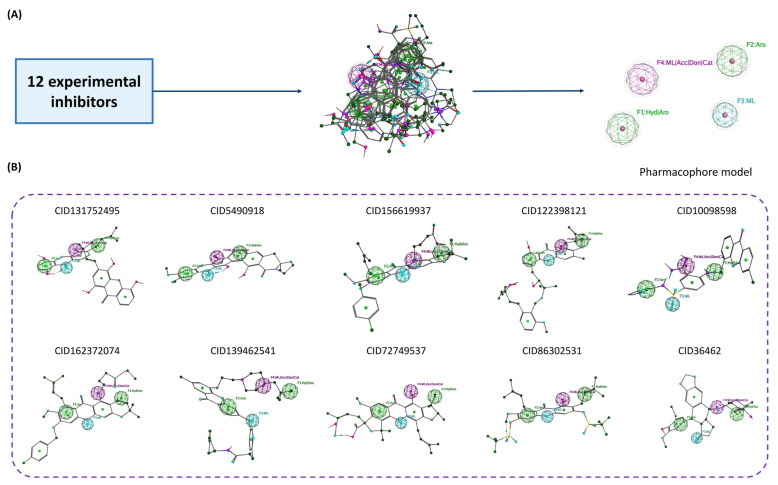
Ligand-based pharmacophore screening. (**A**) Pharmacophore model from 12 experimental inhibitors; (**B**) top 10 representative cluster compounds satisfying the pharmacophore model.

After structural clustering, ligand-based pharmacophore screening was applied to 18 cluster representatives using pharmacophore models constructed from 12 experimentally validated TOP2A inhibitors. Twelve experimental inhibitors were aligned and analyzed using the PCH_all scheme, which includes multiple chemical features of a molecule, to identify similarities in binding mechanisms. The pharmacophore consensus results showed that four structural fragments (Hyd|Aro, Aro, ML, and ML|Acc|Don|Cat) had the highest occurrence rates, with F1 and F2 accounting for 83%, F3 for 75%, and F4 for 67% of the total compounds (Figure 5A). These features reflect key interactions that are conserved across the experimental inhibitors set, including hydrophobic interactions, π–π bonds, hydrogen bonds, and interactions with proton acceptor/donor sites. Only 10 of the 18 cluster representatives, including the reference compound etoposide, fit the pharmacophore model from the experimental inhibitors (Figure 5B). This ensured that the ten compounds selected for further study not only achieved good docking scores but also matched interaction characteristics, providing a solid foundation for subsequent molecular dynamics and molecular pulling simulations.

#### 2.2.4. MD and SMD Evaluation

##### Identifying the HIT Compound

Molecular dynamics (MD) and steered-molecular dynamics (SMD) simulations with similar computational parameters to the method validation study were performed on ten compounds obtained from the ligand-based pharmacokinetic screening. The convergence of the MD simulation trajectories was assessed using the root mean square deviation (RMSD) plot (Figure S6), showing that the trajectories were equilibrated after 50 ns of simulation. In the SMD simulation, similar to the experimental materials, the pulling forces of the top 10 compounds increased rapidly, reaching a maximum value at around 200 ps before rapidly returning to zero, indicating that the dissociation process was complete (see Figure S7). Based on the linear regression Equation (1) calculated in the method validation study, the binding free energy (ΔG_FPL_) was calculated. The maximum work (W) and average force (F_max_) for each compound are detailed in Table 3. Among these, only one compound, CID156619937, exhibited better work and force than the reference inhibitor etoposide; thus, this molecule was identified as a HIT compound.

**Figure 6:**
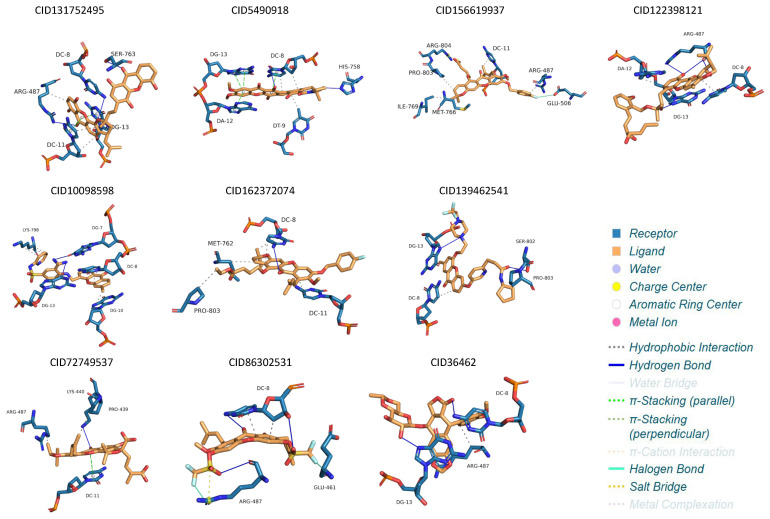
MD refined structure binding pose between the top nine compounds and etoposide on TOP2A. DG, DC, DT, and DA are nucleotides in which DA: adenine, DT: thymine, DG: guanine, and DC: cytosine.

The binding free energy (ΔG_FPL_) values derived from the FPL calculations showed a linear correlation with the number of interactions formed between the ligand and the active site. The binding poses for the top ten compounds are shown in Figure 6.

##### Expand the Search for Analogs in HIT Compound’s Cluster

**Figure 7:**
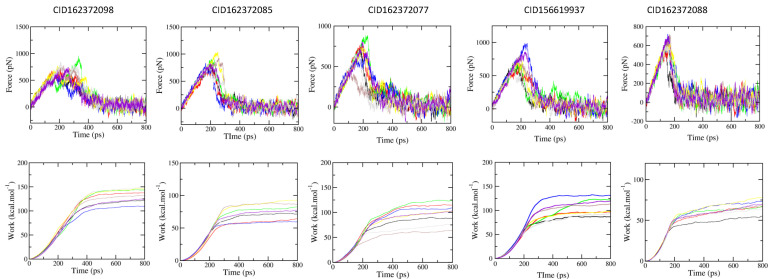
The mean force and work of five complexes during eight independent SMD trajectories.

To analyze the structure–activity relationship and investigate whether the analog scaffolds could maintain the observed mechanical stability, four additional analogs from the same cluster as the HIT compound were subjected to molecular dynamics (MD) and steered molecular dynamics (SMD) analysis. Figure 7 illustrates the mean work and force for the HIT compound, CID156619937, and the four analogs across eight independent SMD trajectories.

The mean maximum force (F_max_) values ranged from 665.8 ± 17.8 pN to 862.5 ± 33.3 pN, while the mean work values ranged from 67.9 ± 2.4 to 133.0 ± 4.9 kcal mol^−1^. The binding energies (ΔG_FPL_) for these five compounds, calculated using the linear regression Equation (1), are presented in Table 4. Among them, analog CID162372098 exhibited the most negative binding affinity of −13.56 kcal mol^−1^. This result confirms that it and the original HIT compound, CID156619937, possess the most favorable predicted binding energy, both significantly surpassing the reference compound, etoposide.

#### 2.2.5. Structural and ADMET Analysis

**Figure 8:**
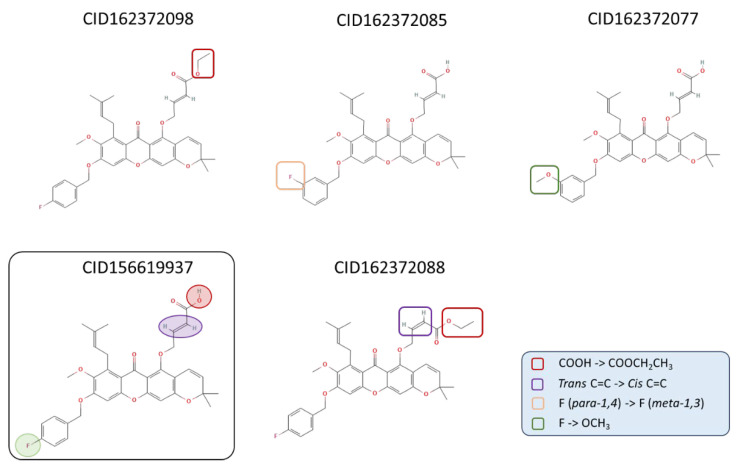
The slight differences in structure among the five analogs.

The structural differences among the selected analogs of cluster 3 were analyzed and are illustrated in Figure 8. Four differences were identified among these compounds, primarily involving functional group substitutions and conformational differences. These differences include modifications of the carboxyl group, positional changes of fluorine substituents, replacement of fluorine with methoxy groups, and cis–trans isomerization within the molecular scaffold. These structural distinctions provided a basis for further comparison of binding characteristics among the compounds.

**Figure 9:**
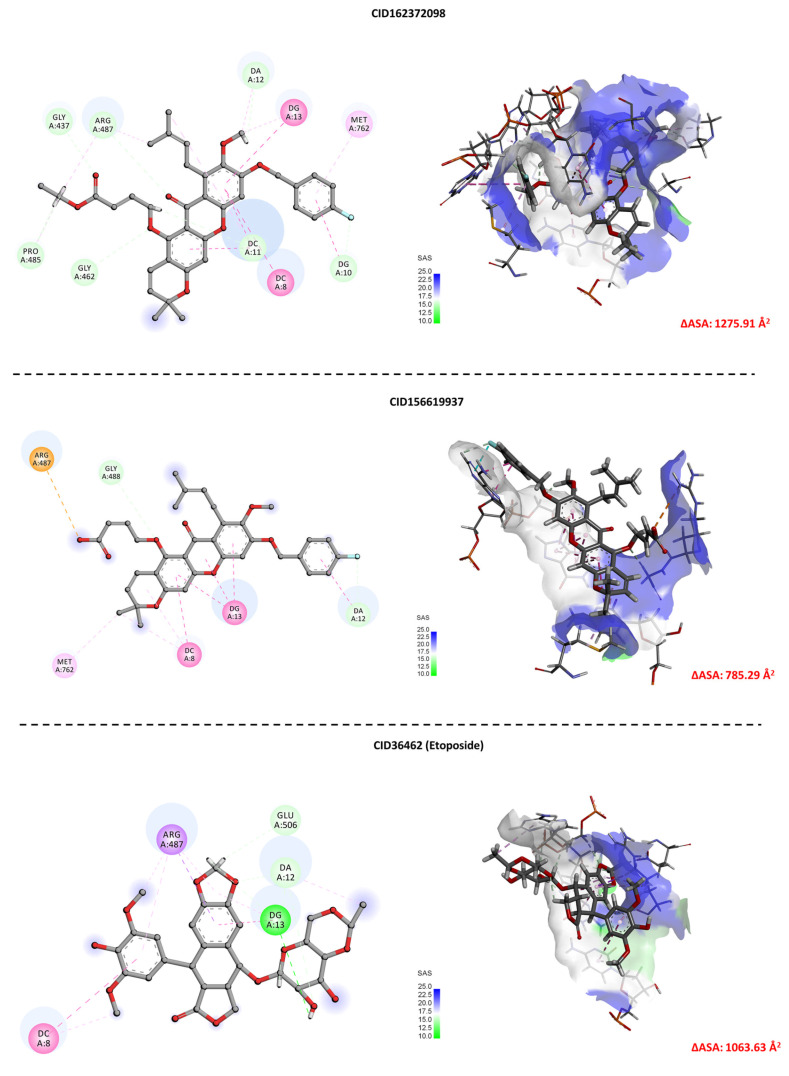
Visualization of ligand–receptor interfaces and solvent-accessible surface area (ΔASA). The top row shows 2D interaction maps highlighting hydrogen bonds and hydrophobic contacts. The bottom row presents the solvent-accessible surface of the receptor with ligand.

To evaluate the extent of protein–ligand interaction, the solvent-accessible surface area (ΔASA) of the complexes was calculated (Figure 9). Binding pocket analysis reveals that CID162372098 exhibits the highest ΔASA value (1275.91 Å^2^), followed by CID156619937 (1063.63 Å^2^) and etoposide (785.29 Å^2^), indicating that CID162372098 occupies the greatest space within the pocket.

This trend is consistent with the enhanced binding affinity observed for these compounds in the FPL simulations. Both CID162372098 and CID156619937 are members of a high-affinity SALI cluster (group 3), where small structural changes lead to large variations in activity. Their structural fragments strongly overlapped the pharmacophore map of known inhibitors while maintaining consistency in the interaction formation with key residues in the TOP2A–DNA complex (Figure S8).

The ADMET properties of the selected compounds are summarized in Table S2.

In terms of absorption, all compounds showed high predicted human intestinal absorption (89–100%), which is higher than that of the reference inhibitor etoposide (75.6%). The Caco-2 permeability values were comparable or slightly improved.

Regarding distribution, all compounds displayed low blood–brain barrier (BBB) permeability (logBB < –1.3), similar to etoposide.

In metabolism, none of the compounds were predicted to inhibit CYP2D6 or CYP3A4 enzymes, while all were predicted to be CYP3A4 substrates.

From a toxicity perspective, most compounds were predicted to be non-AMES toxic and non-hepatotoxic. All compounds showed no hERG I inhibition, although some analogs exhibited potential hERG II inhibition.

#### 2.2.6. Cell Viability Study

The two HIT compounds, CID162372098 and CID156619937, were identified from the ChemSpace library and ordered for cytotoxicity assays. The A549 cell line (human lung adenocarcinoma epithelial cell) and HepG2 (Hepatocellular carcinoma) were selected as the target for evaluation of the cytotoxicity of the compounds using the dimethylthiazol-diphenyltetrazolium bromide (MTT) assay. The control cells showed high proliferation, which could be considered as 100%. Cells were treated with the tested compounds at various concentrations for 48 h.

The cytotoxic activity of the potential HIT compounds, CID162372098 and CID156619937, was evaluated against A549 and HepG2 cell lines (Table 5). It is observed that both compounds exhibit strong proliferation inhibition against the HepG2 cell line, with IC_50_ values of 9.54 ± 0.26 µg mL^−1^ and 12.69 ± 0.31 µg mL^−1^, respectively. Meanwhile, these molecules showed less activity against the A549 cancer cell line, of which the IC_50_ values were recorded at 37.56 ± 0.83 µg mL^−1^ and 49.11 ± 1.16 µg mL^−1^ for CID162372098 and CID156619937.

## 3. Discussion

The present study employed an integrated computational workflow combining molecular docking, molecular dynamics (MD), steered molecular dynamics (SMD), clustering, and pharmacophore modeling to identify potential TOP2A inhibitors from a xanthone compound library. The reliability of this approach is supported by the agreement observed between computational predictions and experimental data in the validation stage.

Among the evaluated docking methods, mVina showed the highest correlation with experimental binding affinities, suggesting its suitability for predicting ligand–protein interactions in the TOP2A system. However, molecular docking provides only a static representation of binding and does not fully capture the dynamic behavior of ligand–protein complexes. To address this limitation, MD simulations were performed to assess structural stability under physiological conditions, showing that all systems reached equilibrium, which allows using a stable configuration for further analysis. In addition, the FPL/SMD approach showed good correlation with experimental data (R = 0.82 ± 0.13), supporting its use for refining binding affinity estimation.

The multi-step screening strategy, including RO5 filtering, clustering, and pharmacophore modeling, efficiently reduced the dataset while maintaining chemical diversity and key interaction features. This approach enabled the identification of representative compounds with favorable binding characteristics.

MD and SMD analyses suggest that CID156619937 is a promising candidate, showing binding characteristics comparable to etoposide and stable interactions with key residues in the TOP2A active site. Further exploration within the same cluster identified CID162372098, which exhibited the most favorable predicted binding affinity among the evaluated compounds.

Notably, analysis of structurally similar analogs within cluster 3 revealed that small chemical modifications may lead to substantial differences in predicted binding behavior. For example, conversion of a carboxyl group to an ethyl ester was associated with improved interactions, whereas geometric changes such as cis–trans isomerization or substituent modifications (e.g., positional changes in halogen atoms or replacement with methoxy groups) were associated with less favorable binding patterns. These structural effects are supported by interaction analysis (Figure S9), which shows that favorable substitutions promote more extensive hydrogen bonding and improved packing within the binding pocket, whereas unfavorable modifications tend to disrupt these interactions. These observations highlight the sensitivity of ligand–protein interactions to subtle structural variations and provide insight into structure–activity relationships within the xanthone scaffold. ΔASA analysis further supports this trend, suggesting that increased accessible surface area in the active site may contribute to stronger ligand–protein interactions.

ADMET predictions suggest that the identified compounds possess generally favorable pharmacokinetic profiles, including high intestinal absorption, low BBB permeability, and minimal predicted CYP inhibition. However, some compounds showed potential hERG II liability or AMES toxicity, indicating that further optimization may be required.

It should be emphasized that the findings of this study are primarily based on computational approaches. While docking, MD, and SMD provide valuable insights into binding modes and stability, these predictions do not necessarily translate directly into biological activity. Therefore, experimental validation remains essential. In this context, in vitro cytotoxicity assays have been conducted to preliminarily evaluate the anticancer activity of the selected HIT compounds. The obtained results showed that CID162372098 and CID156619937 possess anti-proliferative properties. In particular, both compounds exhibit significant cytotoxicity activities against the HepG2 cell line (9.54 ± 0.26 µg mL^−1^ and 12.69 ± 0.31 µg mL^−1^, respectively) and moderate activities against the A549 cell line. These findings support the reliability of our computational screening approach and identify the two HITs as strong candidates for further mechanistic investigation.

## 4. Materials and Methods

### 4.1. Computational Materials

#### 4.1.1. Ligand Preparation

A dataset of 3000 compounds with xanthone scaffold (CID7020) was collected directly from the PubChem database (https://pubchem.ncbi.nlm.nih.gov, accessed on 2 October 2025). Both 2D (two-dimensional) and 3D (three-dimensional) structures of ligands were generated from the SMILES strings using Open Babel 3.0.0. The output structures were then converted to PDBQT format using MGL Tools 1.5.6 for the docking procedure [23].

Ligand protonation states were determined according to their predominant microspecies at physiological pH using Chemicalize web server (chemicalize.com/app, accessed on 29 October 2025) prior to parameterization. The ligand geometries were subsequently optimized at the B3LYP/6-31G(d,p) level of theory using Gaussian 09. Electrostatic potentials were computed employing the Merz–Kollman approach, and atomic partial charges were obtained through RESP fitting.

Force field parameters for the ligands were assigned using the General Amber Force Field (GAFF) implemented in AmberTools18 [32]. Any missing bonded parameters were generated with parmchk2, and topology files were constructed using tleap to produce AMBER prmtop and inpcrd files. Finally, these topologies were converted into GROMACS-compatible format using ACPYPE for subsequent molecular dynamics simulations.

#### 4.1.2. Protein Preparation

The crystal structure of human topoisomerase II alpha (PDB ID: 5GWK) was obtained from the Protein Data Bank “https://www.rcsb.org (accessed on 3 October 2025)”. The co-crystallized ligand (etoposide) and waters were excluded from the structure using PyMOL 1.3 to make it a free receptor. In the next stage, the enzyme was prepared using MGL Tools 1.5.6.

### 4.2. Computational Methods

#### 4.2.1. Molecular Docking Simulation

The docking simulation was performed using modified Vina (mVina), which has been shown to provide better ligand binding affinity ranking than Vina 1.2 [21], thereby enhancing the correlation coefficient between docking energy and experimental results [20]. In addition, Autodock Vina 1.1.2 versions using the empirical scoring function and Autodock Vina using the Vinardo scoring function [22] and the AutoDock4 scoring function [23] were also used to predict the binding affinity between the experimental ligand and the protein. Among them, the mVina method, giving the best correlation, was selected as the molecular docking method for the xanthone dataset [22,23,33]. The docking grid box dimensions for mVina were set to 38 Å × 24 Å × 24 Å, covering the whole active site of TOP2A. The center of the grid box was defined by x, y, and z coordinates of 31.5, −22.0, and −59.0, respectively. Docking simulations were conducted with a global search exhaustiveness setting of 8. The docking conformation with the lowest binding free energy was selected to analyze the interaction mechanism between ligand and TOP2A.

#### 4.2.2. Assessment of Druglikeness

The concept of drug-likeness was developed to identify compounds with properties suitable for drug development, a process that has become increasingly regulated. An important tool for evaluating drug-likeness is Lipinski’s “Rule of Five” [34]. A compound is considered to have drug-like properties if it generally responds to these criteria: molecular weight less than 500 Daltons, high lipophilicity (LogP below 5), no more than 5 hydrogen bond donors, no more than 10 hydrogen bond acceptors, and molar refractivity between 40 and 130. Compounds satisfied with less than 3 criteria of these rules are assumed to have low drug-like potential. The SwissADME web server (http://www.swissadme.ch/index.php, accessed on 21 October 2025) is used to assess the druglikeness of compounds.

#### 4.2.3. Structural Clustering

Clustering analysis was performed on a dataset including xanthone compounds, which was identified based on a drug likeness assessment. The analysis utilized DataWarrior software (version v06.01.00) with the primary objective of grouping structurally similar xanthone derivatives to determine chemical structure in the dataset [31].

In this analysis, the molecular structures were represented by fragment fingerprints (FragFP), a commonly used descriptor for similarity calculations in cheminformatics based on the SMILES of each compound. The clustering process was conducted using DataWarrior’s hierarchical clustering algorithm, which generates a dendrogram based on the calculated Tanimoto similarity between the FragFPs of the compounds [35]. The algorithm is designed to group molecules based on a Tanimoto similarity of at least 80% of their FragFPs, ensuring that compounds within the same cluster have a high degree of structural similarity. This process results in the formation of distinct clusters, with each cluster representing a set of xanthone compounds exhibiting high internal structural similarity.

After cluster formation, the representative compound for each cluster is identified by DataWarrior’s visualization. The representative compound for each cluster can be conceptually understood as the compound whose FragFP is the most geometrically central in the cluster (analogous to a centroid) or the compound that exhibits the highest average Tanimoto similarity to all other FragFPs in its respective cluster (analogous to a medoid) [31]. This approach allows for the identification of a characteristic structural motif or a typical example for each delimited group.

#### 4.2.4. Pharmacophore Modeling

Molecular Operating Environment (MOE version 2022.02) is software developed by Chemical Computing Group, UCL. MOE supports drug design through molecular simulation and protein structure analysis, based on SVL and the Science vector language (Group 2019). When receptor geometry is unknown, pharmacophore modeling in MOE is an efficient way to generate and use 3D geometric data to search for new active compounds. The pharmacophore approaches avoid the structural or chemical class bias of 2D approaches by using a generalized ligand representation and geometric limitations. The pharmacophore model was built on 12 experimental inhibitors evaluated by bioassay on TOP2A, some of which have been approved as commercial drugs for cancer treatment.

#### 4.2.5. Molecular Dynamics Simulation

GROMACS version 2019.6 [36] software will be used to simulate the structural changes in the TOP2A-ligand complex in solution. In which the TOP2A enzyme and ions are parameterized by the Amber99SB-iLDN force field [37]. The ligands will be described using the generalized Amber force field (GAFF) [32]. Water molecules are represented by the TIP3P model [38]. The TOP2A-ligand complex is then placed in a cubic with complete boundary conditions, maintaining a minimum distance of 1.2 nm between the complex and the box walls. Sodium (Na^+^) and chloride (Cl^−^) ions will be added to neutralize the overall charge of the solvated system. Electrostatic interactions will be calculated using the Particle Mesh Ewald (PME) method [39], while Van der Waals interactions will be treated with a cutoff distance of 0.9 nm. The solvation system will be minimized by steepest descent, and then the system will be recovered in 100 ps of position-constrained simulations in NVT (number of particles, volume, and temperature) and NPT (number of particles, pressure, and temperature) representations at 310 K. Then, three molecular dynamics (MD) simulations of 100 ns duration will be performed to obtain the stable configuration of the complex. The coordinates of the solvated system will be saved every 10 ps for subsequent analysis.

#### 4.2.6. Steered Molecular Dynamics Simulation

**Figure 10:**
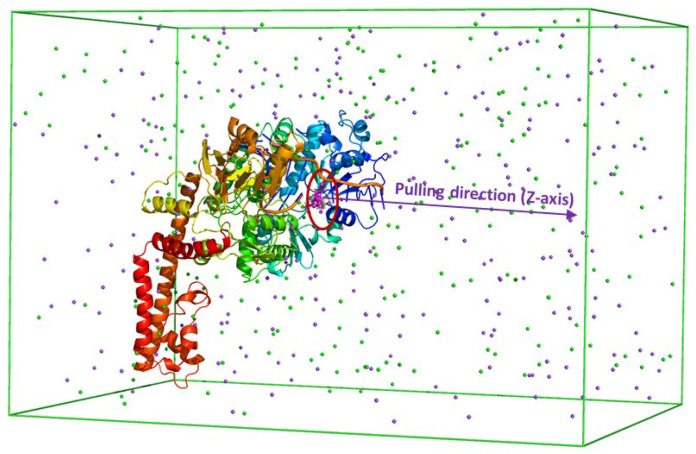
SMD illustration of a complex along the *z*-axis.

Representative cluster structures of the topoisomerase II alpha inhibitor (TOP2A) complex, obtained from the analysis of three independent molecular dynamics (MD) trajectories, served as the starting configuration for subsequent steered-molecular dynamics (SMD) simulations. The complexes were put into a triclinic box, and the dissociation of the inhibitor from the active site of topoisomerase II alpha was induced by applying an external harmonic potential. The TOP2A-Ligand complex was placed in a rectangular PBC (periodic boundary conditions) box with full boundary conditions. Na^+^/Cl^−^ ions were added to neutralize the solvated system. Electrostatic interactions were calculated using the particle Ewald electrostatic method [39]. The van der Waals interactions between the particles were effective within 0.9 nm. The solvated system is minimized by the steepest descent, and then the system is recovered in 100 ps of position-restricted simulations in NVT (number of particles, volume, and temperature) and 600 ps NPT (number of particles, pressure, and temperature) representations at 310K. Finally, the ligand was pulled out of the TOP2A binding pocket by applying the spring constant (v) of the cantilever, and the pulling velocity (k) was set to 600 kJ mol^−1^ nm^−2^ and 0.005 nm ps^−1^, respectively (Figure 10). During the SMD simulation, the ligand displacement and pulling force were recorded every 0.1 ps to estimate the ligand binding affinity. In total, the FPL calculations were performed independently eight times to ensure adequate sampling.

#### 4.2.7. Computational Analysis Tools

The 2D interaction diagrams between TOP2A inhibitors were illustrated via the BIOVA Discovery studio, and 3D interaction was generated using Protein-Ligand Interaction Profiler (PLIP) web tools [40]. ChemAxon webserver (www.chemicalize.com, accessed on 18 November 2025) was used to predict the protonation state of the ligand. The GROMACS “gmx rms” tool calculates the backbone RMSD between the configuration of the complex before and after MD refinement [41]. The pkCSM “https://biosig.lab.uq.edu.au/pkcsm (accessed on 21 October 2025)”, a powerful and user-friendly web tool designed for the in silico, was utilized to predict the ADMET properties of a potential compound by simply inputting a chemical structure’s SMILES notation (https://pubchem.ncbi.nlm.nih.gov, accessed on 23 October 2025) [42]. The solvent-exposed surface area (ASA) of each complex was calculated using the FreeSASA program [43], applying the Shrake–Rupley algorithm with a probe radius of 1.4 Å. The solvent-exposed surface area (ΔASA) upon binding was calculated using the formula ΔASA = (ASA_protein + ASA_ligand) − ASA_complex.

### 4.3. Biological Experiments

#### 4.3.1. Cell Lines and Cell Culture

The A549 (Human lung adenocarcinoma epithelial cell) and HepG2 (Hepatocellular carcinoma) cell lines were obtained from ATCC (Manassas, VA, USA) and maintained at 37 °C in 5% CO_2_ in suitable media (RPMI 1640, MEM, DMEM; Merck KGaA, Darmstadt, Germany) containing 10% heat-inactivated fetal bovine serum (FBS), penicillin (100 UI.mL^−1^), streptomycin (100 mg mL^−1^), and Lglutamine (2 mM).

#### 4.3.2. Cytotoxic Assay

Cell viability was assessed through MTT [3-(4,5-dimethylthiazol-2-yl)-2,5-diphenyltetrazolium bromide] assay [44,45] was adopted to measure the in vitro cytotoxicity of the nanoparticles in the cancer cell lines. Briefly, the cells were seeded in a 96-well plate (5 × 10^4^ cells per well of 200 µL mixture) and attached for 24 h. The cells were subsequently treated with two HIT compounds in the range 100 to 1 μg mL^−1^ and incubated at 37 °C and 5% CO_2_ for 48 h. The control was selected as ellipticine. At the end of incubations, 20 μL of MTT (Merck KGaA) was added to the wells and incubated at 37 °C for 4 h. Absorbance was recorded at 540/720 nm by using a Spark multimode reader (Tecan, Männedorf, Switzerland). The growth inhibition was assessed using the following formula: inhibition rate (%) = (1 − OD_sample_/OD_con_) × 100%, where OD_sample_ and OD_con_ are the optical densities of the experimental sample groups and control, respectively. The aforementioned experiment was carried out three times to get average IC_50_ values using TableCurve AISN Software (Jandel Scientific, San Rafael, CA, USA). Cytotoxicity is expressed as an IC_50_ value (the concentration of test agents that cause 50% inhibition or cell death), which was obtained by nonlinear regression using the TableCurve 2D version 5.01 program (SPSS Statistical Software, Chicago, IL, USA).

## 5. Conclusions

In this study, two xanthone derivatives, CID162372098 and CID156619937, were identified as promising TOP2A inhibitors through molecular docking and steered molecular dynamics simulation. CID162372098 presented the most favorable binding free energy (ΔG_FPL_ = –13.60 kcal.mol^−1^; ΔG_mVina_ = –15.6 kcal.mol^−1^), benefited from an ethyl ester substitution that optimizes pocket fit, extends hydrogen-bonding and preserves key interactions. CID156619937 (ΔG_FPL_ = –10.76 kcal.mol^−1^; ΔG_mVina_ = –15.7 kcal.mol^−1^) retained a strong hydrogen-bond network and π–stacking with essential residues, supporting its role as a potential backup HIT. Both compounds showed stable binding in the binding pocket of the protein and maintained the pharmacophore features of known TOP2A inhibitors. In addition, ADMET predictions further suggest acceptable drug-like properties, supporting their potential for oral administration. Our preliminary cytotoxicity assay showed CID162372098 and CID156619937 as effective anti-proliferative molecules against the HepG2 cell line. In general, these studies establish a strong basis for further mechanistic investigations and support the development of effective TOP2A inhibitors for cancer therapy.

## Figures and Tables

**Table 1:** Molecular docking results of experimental inhibitors by different methods.

No.	ChemID	ΔG_Exp_ ^a^	ΔG_mVina_ ^b^	ΔG_Vina1.1.2_ ^b^	ΔG_Vinardo_ ^b^	ΔG_AD4_ ^b^
1	CID65907	−12.11	−15.6	−8.9	−5.93	−11.76
2	CID42890	−11.78	−14.8	−8.2	−6.03	−11.38
3	CID30323	−10.98	−14.5	−8.8	−5.43	−11.15
4	CID400718	−9.91	−14.2	−9.4	−5.84	−10.75
5	CID76309460	−9.44	−13.4	−8.7	−6.35	−8.45
6	CID155186428	−9.19	−11.8	−8.1	−5.63	−7.63
7	CID31703	−8.54	−14.6	−8.3	−5.55	−11.47
8	CID2179	−8.43	−12.3	−7.6	−6.08	−6.92
9	CID122192946	−6.86	−11.5	−8.0	−6.01	−8.84
10	CID155525473	−6.38	−13.0	−8.1	−5.86	−7.77
11	CID72707984	−8.29	−14.5	−8.8	−5.53	−8.99
12	CID189219	−8.04	−12.3	−8.8	−6.64	−7.22
**Correlation**			0.70±0.16	0.41±0.21	−0.13±0.21	0.70±0.15

^a^ The experimental binding free energy ΔG_exp_ was computed via the reported IC_50_ on the binding database, with the supposition that the IC_50_ value is equal to the inhibition constant K_i_, the unit is in kcal. mol^−1^; ^b^ the docking affinity was gained using the Autodock package. SE of Pearson correlation estimated via nonparametric bootstrap resampling.

**Table 2:** The obtained values of the FPL calculations in comparison with the respective experiments.

No.	PubChem ID	F_max_ ^a^	W ^b^	ΔG_FPL_ ^c^	ΔG_exp_ ^d^
1	CID65907	726.2 ± 42.9	108.4 ± 6.7	−10.84	−12.11
2	CID42890	985.9 ± 61.6	130.9 ± 10.4	−13.33	−11.78
3	CID30323	639.0 ± 66.2	92.6 ± 10.4	−9.09	−10.98
4	CID400718	848.6 ± 20.8	112.4 ± 6.4	−11.29	−9.91
5	CID76309460	704.8 ± 54.6	89.6 ± 8.3	−8.76	−9.44
6	CID155186428	679.7 ± 56.4	91.4 ± 6.9	−8.96	−9.19
7	CID31703	697.6 ± 56.2	94.1 ± 8.0	−9.25	−8.54
8	CID2179	691.9 ± 30.2	106.9 ± 4.4	−10.68	−8.43
9	CID122192946	501.9 ± 38.2	68.4 ± 4.4	−6.42	−6.86
10	CID155525473	507.8 ± 42.8	58.1 ± 6.2	−5.27	−6.38
11	CID72707984	615.7 ± 50.5	86.2 ± 8.4	−8.38	−8.29
12	CID189219	716.5 ± 71.8	80.0 ± 6.1	−7.7	−8.04
**Correlation**		0.72 ± 0.18	0.82 ± 0.13		

^a^ The obtained value of the mean rupture force, Fmax (pN); ^b^ the record metric of the pulling work, W (kcal mol^−1^); ^c^ predicted binding free energy can be calculated via Equation (1), ΔG_FPL_ ^c^ (kcal mol^−1^); ^d^ the binding free energy was computed via IC_50_ value (kcal mol^−1^).

**Table 3:** Calculated binding affinity through SMD for the top nine compounds and etoposide on TOP2A.

No.	PubChem ID	F_max_ ^a^	W ^b^	ΔG_FPL_ ^c^	ΔG_mVina_ ^d^
1	CID131752495	720.1 ± 29.6	82.6 ± 3.2	−7.98	−16.5
2	CID5490918	909.0 ± 30.9	90.4 ± 2.7	−8.85	−15.6
3	CID156619937	771.8 ± 37.5	107.8 ± 5.7	−10.77	−15.7
4	CID122398121	829.8 ± 30.5	86.0 ± 3.2	−8.37	−16.7
5	CID10098598	510.7 ± 31.7	58.1 ± 3.9	−5.27	−16.2
6	CID162372074	656.7 ± 23.9	70.2 ± 1.4	−6.62	−16.4
7	CID139462541	928.8 ± 46.8	100.6 ± 5.0	−9.97	−17.4
8	CID72749537	379.2 ± 19.9	33.5 ± 3.6	−2.55	−16
9	CID86302531	844.9 ± 36.9	71.3 ± 3.8	−6.74	−15.9
10	CID36462 (etoposide)	723.3 ± 36.3	105.9 ± 4.6	−10.56	−15.5

^a^ The obtained value of the mean rupture force, Fmax (pN); ^b^ the record metric of the pulling work, W (kcal mol^−1^); ^c^ predicted binding free energy can be calculated via Equation (1), ΔG_FPL_ ^c^ (kcal mol^−1^); ^d^ predicted binding free energy calculated by mVina molecular docking ΔG_mVina_ (kcal mol^−1^); the computed error is the standard error of the mean of eight independent trajectories.

**Table 4:** Calculated binding affinity through SMD for HIT compounds and their analog on TOP2A.

No.	PubChem ID	F_max_ ^a^	W ^b^	ΔG_FPL_ ^c^	ΔG_mVina_ ^d^
1	CID162372098	771.3 ± 33.3	133.0 ± 4.9	−13.56	−15.6
2	CID162372085	862.5 ± 33.3	76.5 ± 3.9	−7.32	−16.1
3	CID162372077	723.0 ± 44.1	97.7 ± 7.1	−9.66	−15.9
4	CID162372088	665.8 ± 17.8	67.9 ± 2.4	−6.36	−16.0
5	CID156619937	771.8 ± 37.5	107.8 ± 5.7	−10.77	−15.7

^a^ The obtained value of the mean rupture force, F_max_ (pN); ^b^ the record metric of the pulling work, W (kcal mol^−1^); ^c^ predicted binding free energy can be calculated via Equation (1), ΔG_FPL_ ^c^ (kcal mol^−1^); ^d^ predicted binding free energy calculated by mVina molecular docking ΔG_mVina_ (kcal mol^−1^); the computed error is the standard error of the mean of eight independent trajectories.

**Table 5:** Cytotoxic activity (IC50, µM) of HIT compounds on HeLa and A549 cell lines after 48 h of incubation.

No.	PubChem ID	A549 ^a^	HepG2 ^a^
1	CID162372098	37.56 ± 0.83	9.54 ± 0.26
2	CID156619937	49.11 ± 1.16	12.69 ± 0.31
3	ellipticine	0.43 ± 0.12	0.37 ± 0.15

^a^ IC_50_ value (µM mL^−1^). Results are expressed as the mean ± SD values from 3 independent experiments.

## Data Availability

The original contributions presented in this study are included in the article. Further inquiries can be directed to the corresponding author.

## Supplementary Materials

The following supporting information can be downloaded at: https://www.mdpi.com/article/10.3390/molecules31101670/s1. Figure S1. Binding pose between the experimental inhibitors on TOP2A after molecular docking. Figure S2. (a). RMSD of experimental inhibitors on the topoisomerase II alpha (PDB ID: 5GWK) along three 100 ns trajectories. (b). RMSF of experimental inhibitors on the topoisomerase II alpha (PDB ID: 5GWK) along three 100 ns trajectories. (c). Radius of gyration of experimental inhibitors on the topoisomerase II alpha (PDB ID: 5GWK) along three 100 ns trajectories. Figure S3. Steered molecular dynamic results of experimental inhibitor. Figure S4. 18 representatives of each cluster are highlighted by circle dots. Figure S5. SALI-based structural similarity network of 18 xanthone clusters. Figure S6. RMSD, RMSF, radius of gyration of top 9 xanthone derivatives and etoposide (CID36462) on the topoisomerase II alpha (PDB ID: 5GWK) along three 100 ns trajectories. Figure S7. Steered molecular dynamic results of top 9 xanthone derivatives and etoposide (CID36462). Figure S8. Pharmacophore model of the HIT compound and four analog compounds. Figure S9. MD refined structure binding pose between the CID156619937 and its analogs on TOP2A. Figure S10. RMSD, RMSF, radius of gyration of the CID156619937 and its analogs on the topoisomerase II alpha (PDB ID: 5GWK) along three 100 ns trajectories. Table S1. Top 32 xanthone derivatives and etoposide satisfy docking and RO5 filtering. Table S2. ADMET prediction for hit compound and its analogs.
