# Supplementary material for: Structure-Based Drug Design Targeting Topoisomerase II Alpha: Discovery of Potential Antitumor Xanthone Derivatives
Source: Molecules. 2026 May 15;31(10):1670. doi: 10.3390/molecules31101670 (PMC13210332; doi:10.3390/molecules31101670)
Supplement: Supplementary file 1 [file molecules-31-01670-s001.zip › Supplementary Information.pdf]

Supplementary Data

Structure-based drug design targeting Topoisomerase II alpha: Discovery of potential antitumor xanthone derivatives

Thi Thuy Huong Le [1,2,†], Thi Nguyet Hang Nguyen [3,†], Minh Quan Pham [1,2], Thi Thu Thuy Tran [1], Thi Tu Dinh [1,2], Thi Hoai Van Tran [4], Van Lang Tran [5] and Quoc Long Pham [6,7,*]

[1] Institute of Chemistry, Vietnam Academy of Sciences and Technology, 18 Hoang Quoc Viet, Nghia Do, Hanoi 100000, Vietnam
[2] Faculty of Chemistry, Graduate University of Science and Technology, Vietnam Academy of Sciences and Technology, 18 Hoang Quoc Viet, Nghia Do, Hanoi 100000, Vietnam
[3] Faculty of Pharmaceutical Chemistry and Technology, Hanoi University of Pharmacy, 13-15 Le Thanh Tong, Cua Nam, Hanoi 100000, Vietnam
[4] Faculty of Basic Sciences, Vietnam University of Traditional Medicine, Ministry of Health, Tran Phu, Dai Mo, Hanoi 100000, Vietnam
[5] Ho Chi Minh City University of Foreign Languages - Information Technology, Ho Chi Minh City 700000, Vietnam
[6] Laboratory of Biophysics, Institute for Advanced Study in Technology, Ton Duc Thang University, Ho Chi Minh City 700000, Vietnam
[7] Faculty of Pharmacy, Ton Duc Thang University, Ho Chi Minh City 700000, Vietnam
* Correspondence: phamquoclong@tdtu.edu.vn; Tel : +84-933951867
† These authors contributed equally to this work

Contents

Figure S1. Binding pose between the experimental inhibitors on TOP2A after molecular docking 3

Figure S2a. RMSD of experimental inhibitors on the topoisomerase II alpha (PDB ID: 5GWK) along three 100 ns trajectories 3

Figure S2b. RMSF of experimental inhibitors on the topoisomerase II alpha (PDB ID: 5GWK) along three 100 ns trajectories 4

Figure S2c. Radius of gyration of experimental inhibitors on the topoisomerase II alpha (PDB ID: 5GWK) along three 100 ns trajectories 5

Figure S3. Steered molecular dynamic results of experimental inhibitor 6

Figure S4. 18 representatives of each cluster are highlighted by circle dots 7

Figure S5. SALI-based structural similarity network of 18 xanthone clusters 7

Figure S6. RMSD, RMSF, radius of gyration of top 9 xanthone derivatives and etoposide (CID36462) on the topoisomerase II alpha (PDB ID: 5GWK) along three 100 ns trajectories 8

Figure S7. Steered molecular dynamic results of top 9 xanthone derivatives and etoposide (CID36462) 9

Figure S8. Pharmacophore model of the HIT compound and four analog compounds 10

Figure S9. MD refined structure binding pose between the CID156619937 and its analogs on TOP2A 10

Figure S10. RMSD, RMSF, radius of gyration of the CID156619937 and its analogs on the topoisomerase II alpha (PDB ID: 5GWK) along three 100 ns trajectories 11

Table S1. Top 32 xanthone derivatives and etoposide satisfy docking and RO5 filtering 12

Table S2. ADMET prediction for hit compound and its analogs 13

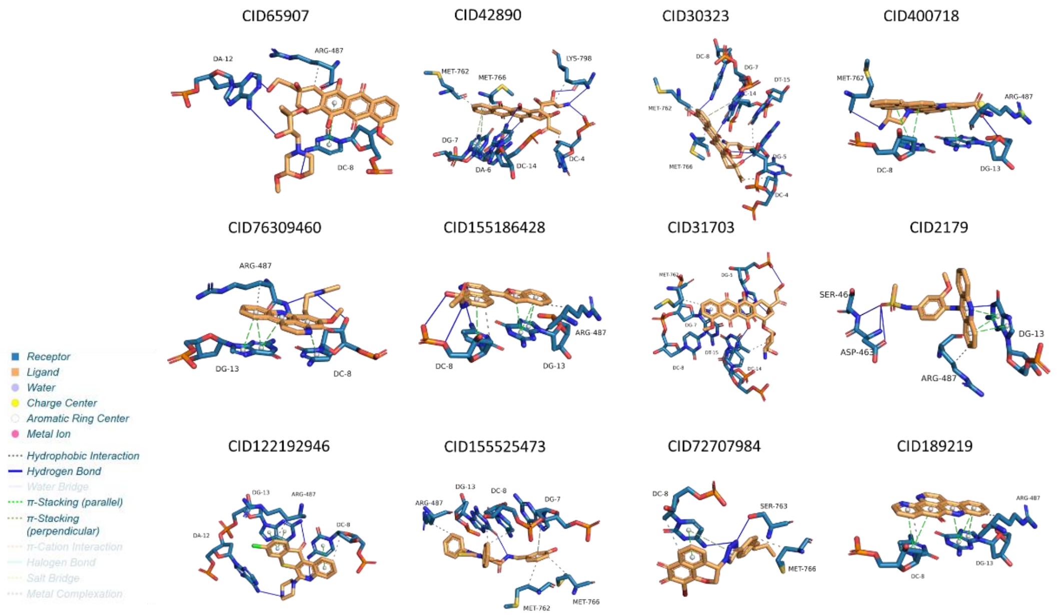

Figure S1. Binding pose between the experimental inhibitors on TOP2A after molecular docking

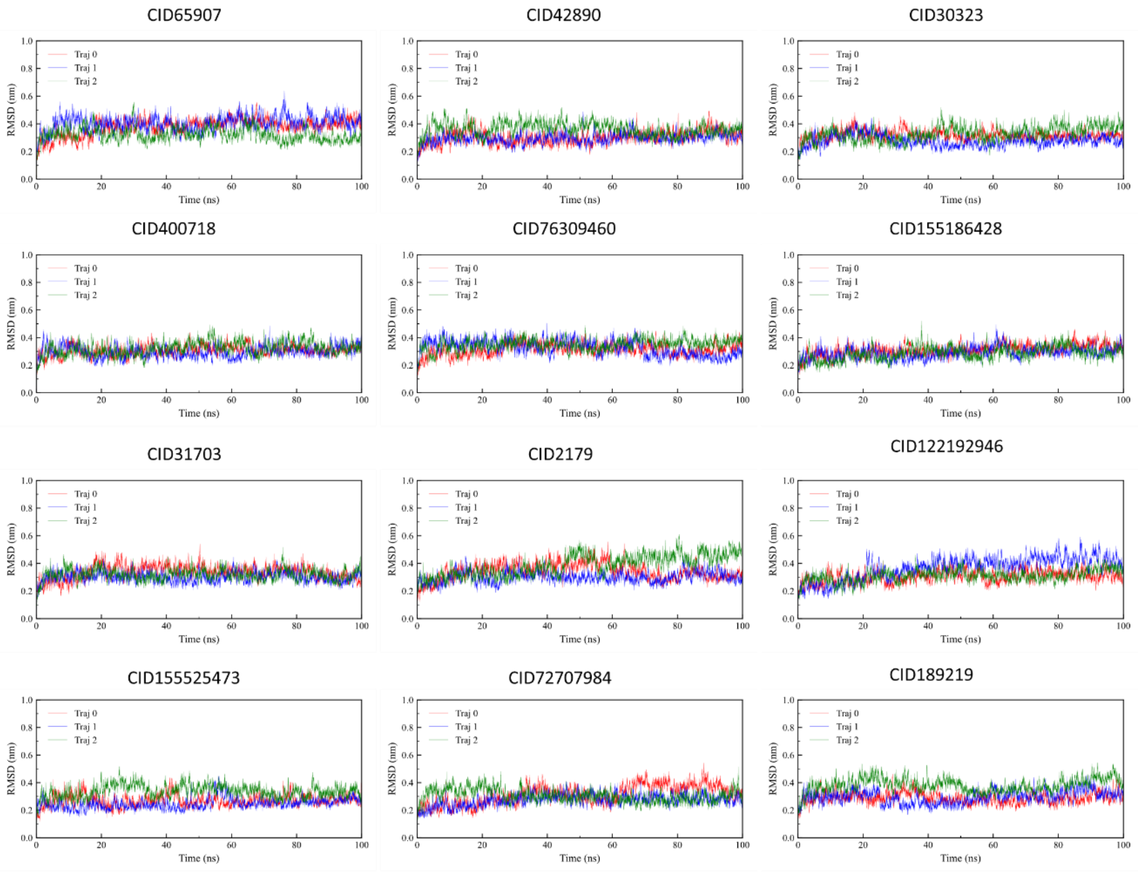

Figure S2a. RMSD of experimental inhibitors on the topoisomerase II alpha (PDB ID: 5GWK) along three 100 ns trajectories

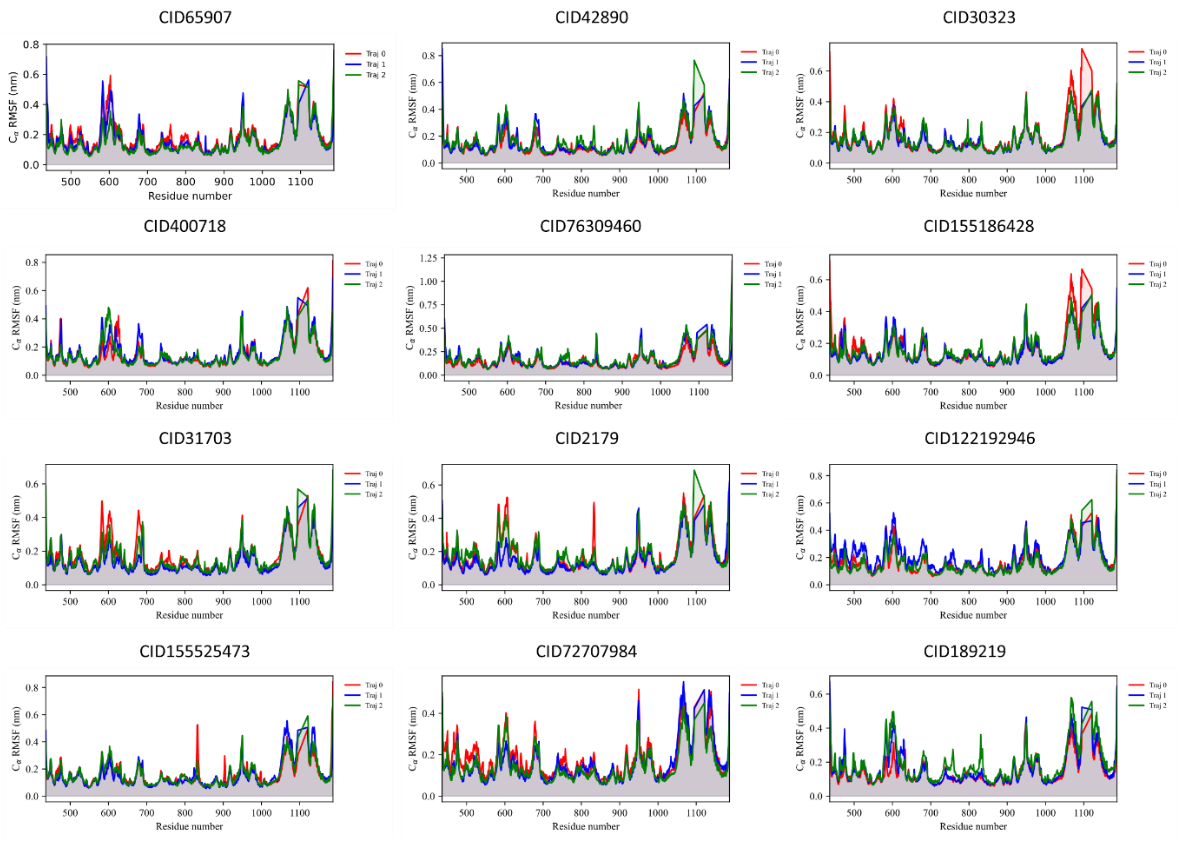

Figure S2b. RMSF of experimental inhibitors on the topoisomerase II alpha (PDB ID: 5GWK) along three 100 ns trajectories

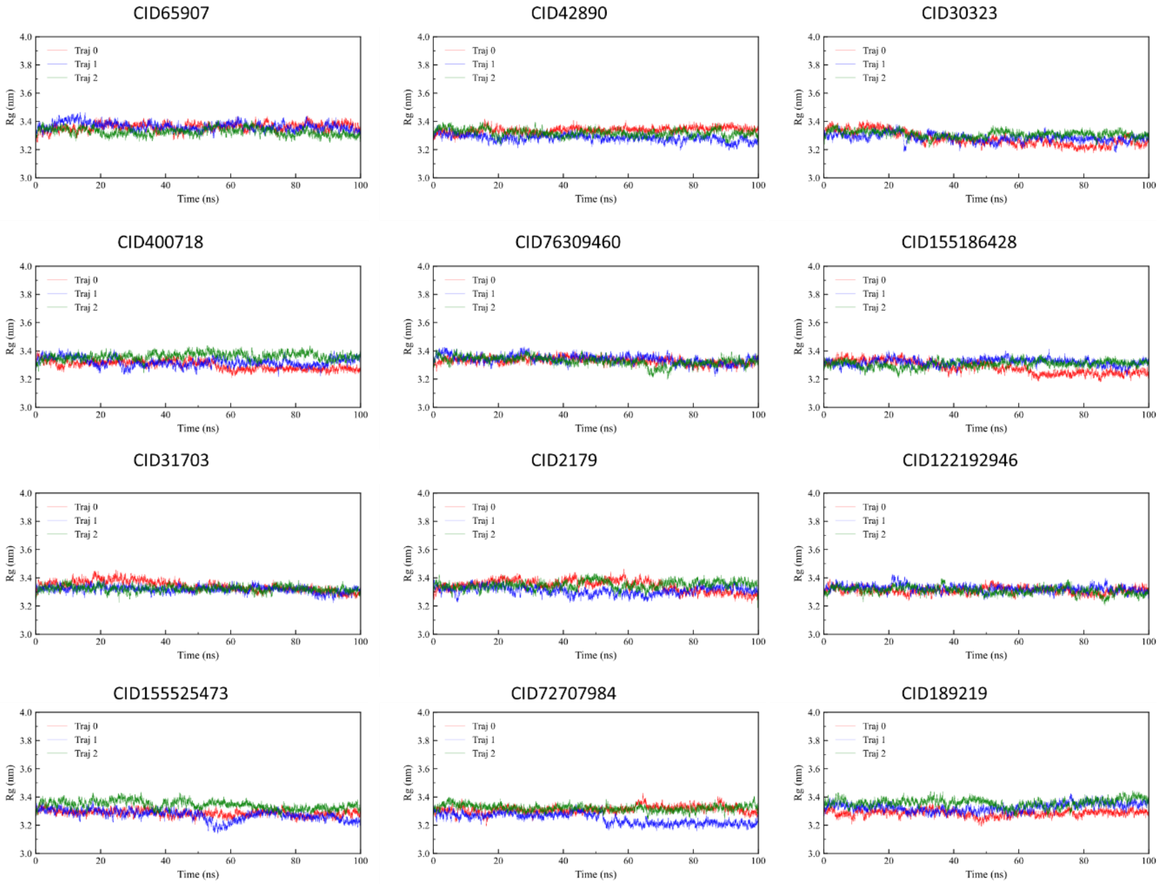

Figure S2c. Radius of gyration of experimental inhibitors on the topoisomerase II alpha (PDB ID: 5GWK) along three 100 ns trajectories

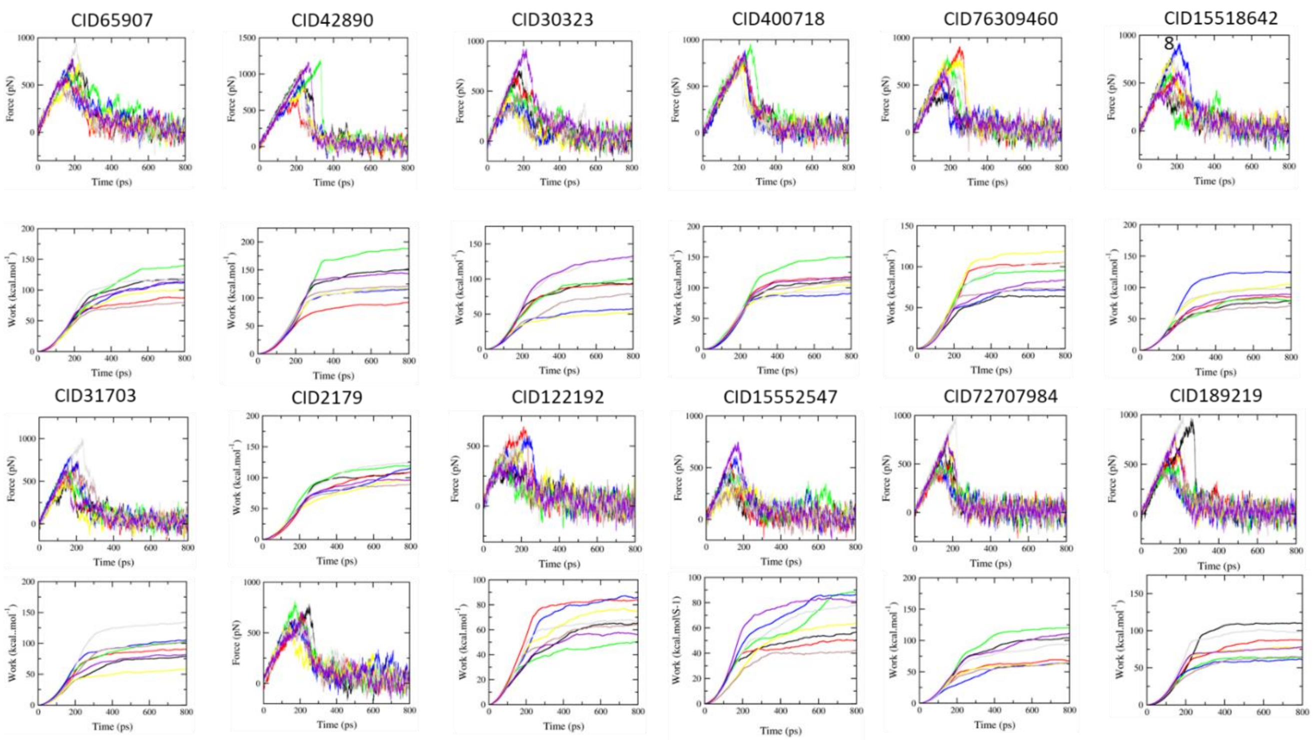

Figure S3. Steered molecular dynamic results of experimental inhibitor

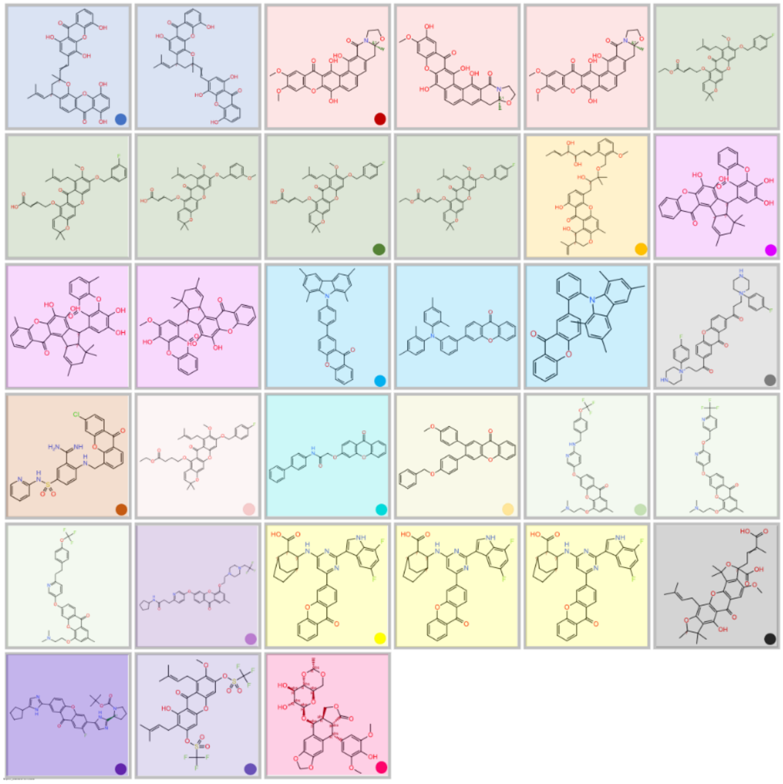

Figure S4. 18 representatives of each cluster are highlighted by circle dots

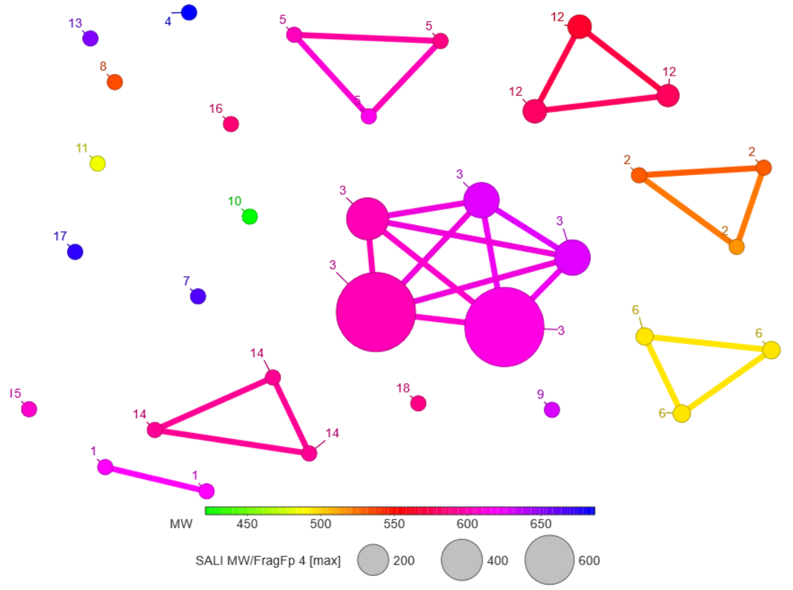

Figure S5. SALI-based structural similarity network of 18 xanthone clusters

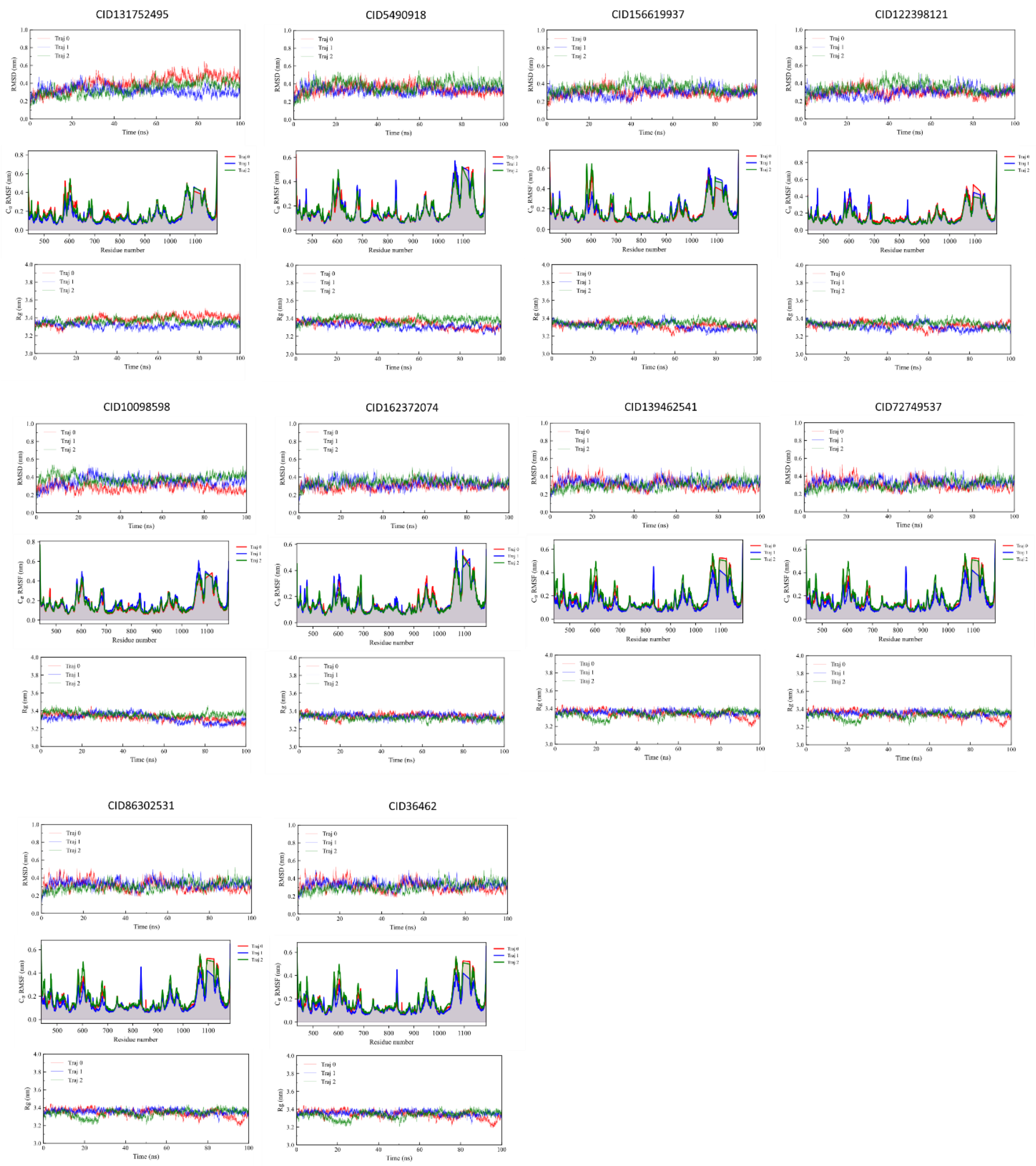

Figure S6. RMSD, RMSF, radius of gyration of top 9 xanthone derivatives and etoposide (CID36462) on the topoisomerase II alpha (PDB ID: 5GWK) along three 100 ns trajectories

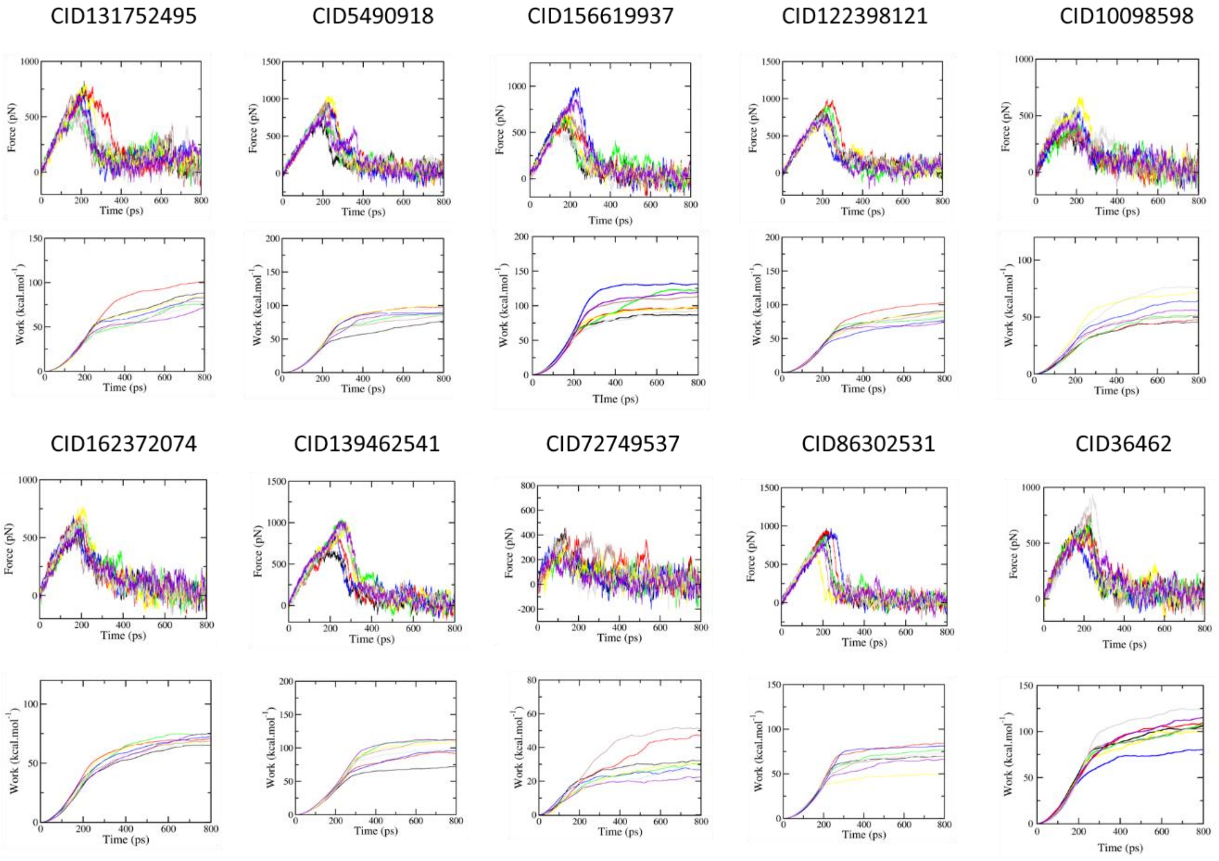

Figure S7. Steered molecular dynamic results of top 9 xanthone derivatives and etoposide (CID36462)

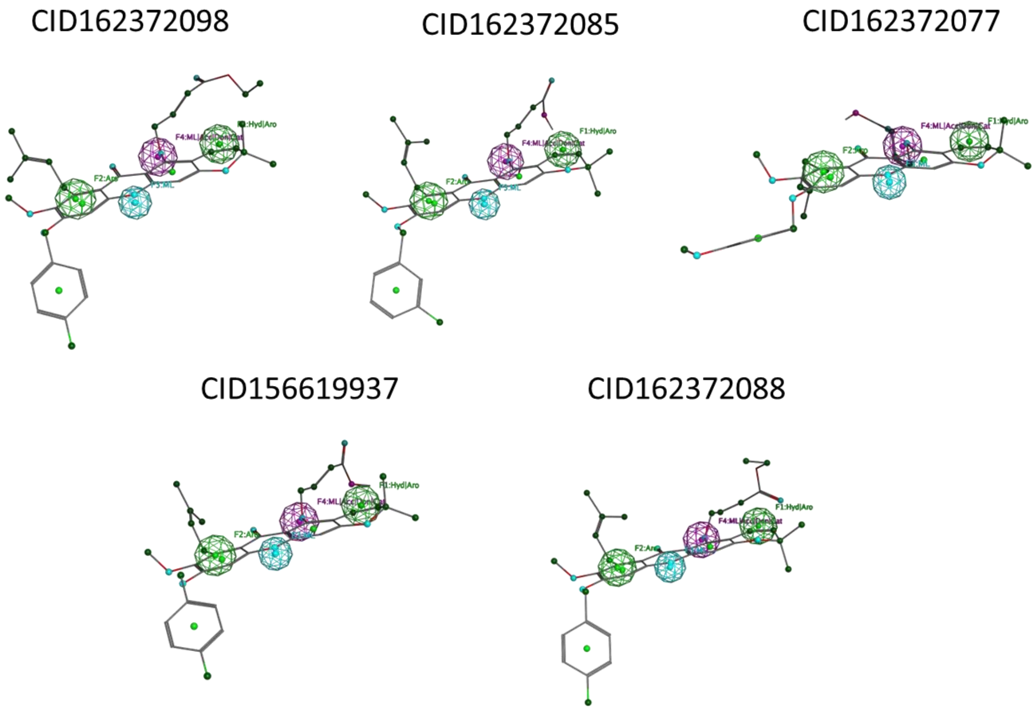

Figure S8. Pharmacophore model of the HIT compound and four analog compounds

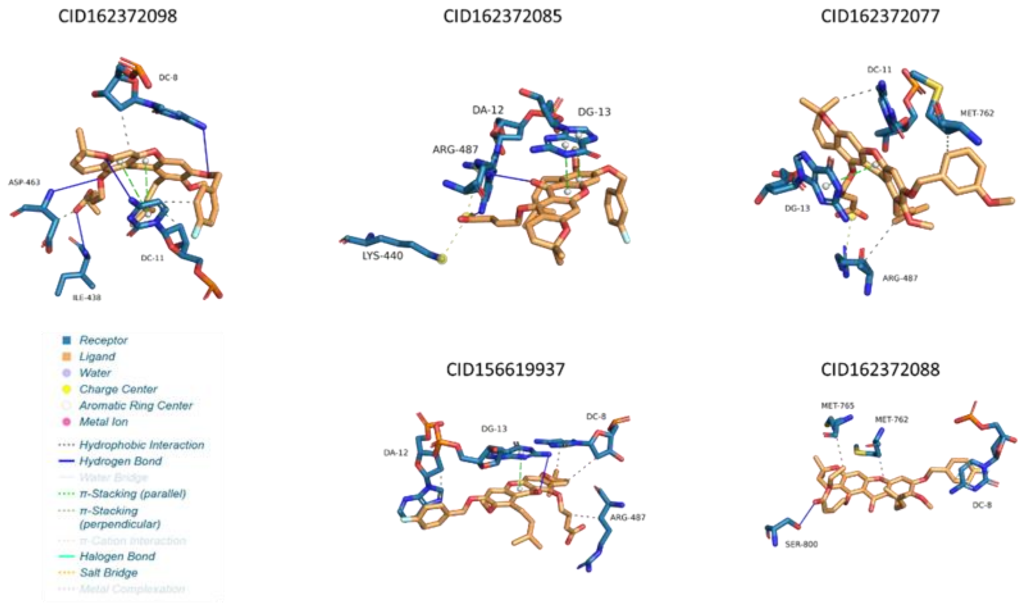

Figure S9. MD refined structure binding pose between the CID156619937 and its analogs on TOP2A

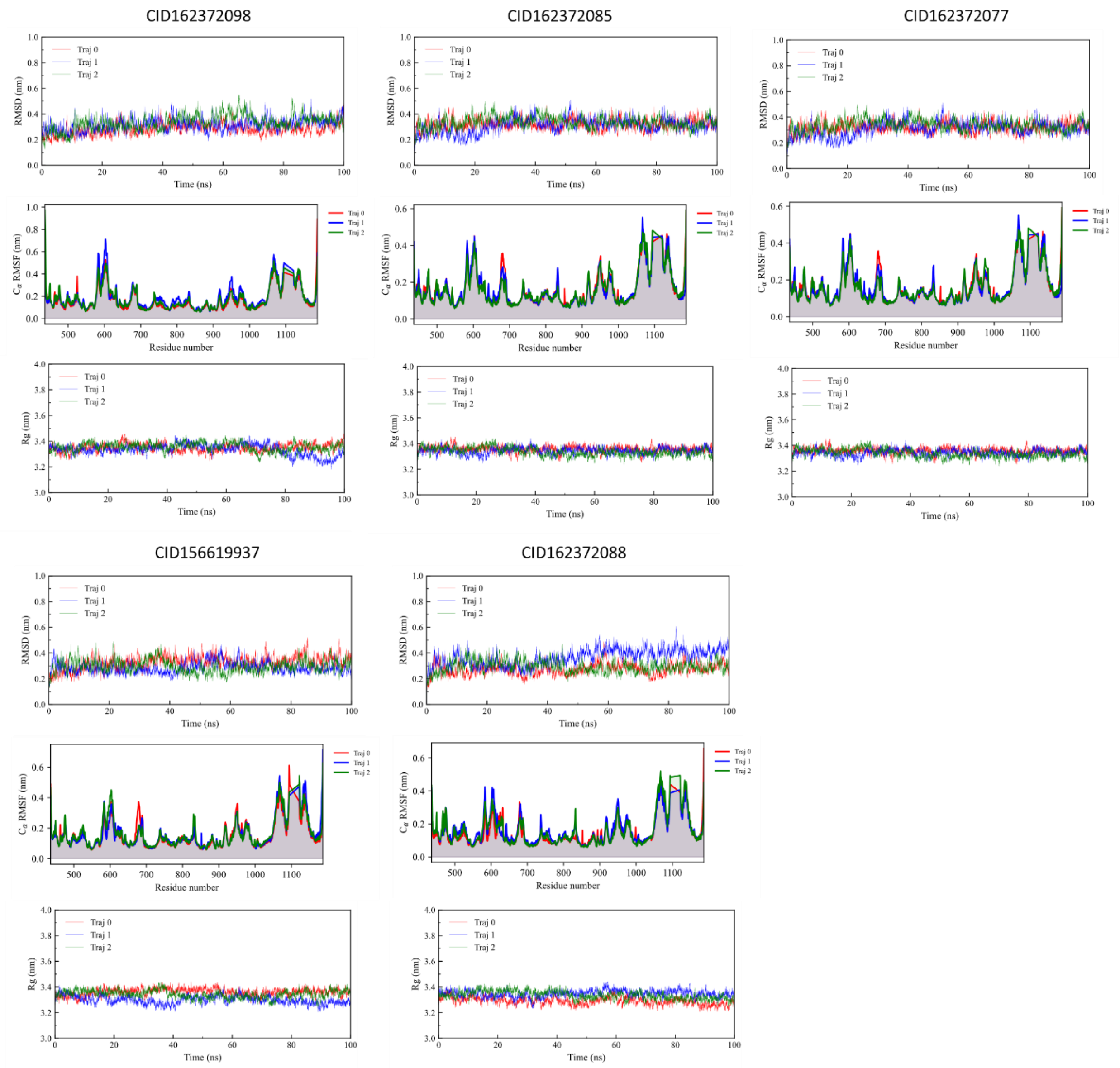

Figure S10. RMSD, RMSF, radius of gyration of the CID156619937 and its analogs on the topoisomerase II alpha (PDB ID: 5GWK) along three 100 ns trajectories

Table S1. Top 32 xanthone derivatives and etoposide satisfy docking and RO5 filtering

No.	PubChem ID	ΔG_{mVina}	MW	nHA	nHD	MLogP
1	CID138747626	-19.2	592.59	8	3	4.15
2	CID134272657	-17.7	592.59	8	3	4.15
3	CID134272656	-17.7	592.59	8	3	4.15
4	CID139462541	-17.4	652.7	11	1	2.22
5	CID145721079	-16.8	515.47	9	4	0.94
6	CID122398121	-16.7	686.79	10	5	1.51
7	CID131752495	-16.5	620.6	10	5	4.62
8	CID165413500	-16.5	616.66	8	4	3.26
9	CID162372074	-16.4	630.7	9	0	3.98
10	CID139462398	-16.3	565.54	11	0	2.21
11	CID89891127	-16.3	583.65	7	2	2.9
12	CID165413463	-16.2	588.6	8	4	2.9
13	CID165413417	-16.2	602.63	8	3	3.08
14	CID10098598	-16.2	533.99	6	4	2.26
15	CID162372085	-16.1	600.63	9	1	3.55
16	CID10326547	-16.1	620.6	10	5	1.83
17	CID139462588	-16.1	579.57	10	1	2.67
18	CID145721078	-16	529.49	9	3	1.13
19	CID165377231	-16	493.59	2	0	5.97
20	CID162372088	-16	628.68	9	0	3.91
21	CID139462301	-16	578.58	10	0	2.97
22	CID72749537	-16	606.66	10	3	2.22
23	CID162372077	-15.9	612.67	9	1	2.87
24	CID86302531	-15.9	674.58	16	1	3.03
25	CID156619937	-15.7	600.63	9	1	3.55
26	CID72946252	-15.7	421.44	4	1	3.14
27	CID5490918	-15.6	529.49	9	3	1.13
28	CID162372098	-15.6	628.68	9	0	3.91
29	CID11540319	-15.6	484.54	4	0	4.58
30	CID165377239	-15.5	495.61	2	0	6.07
31	CID18008579	-15.5	666.76	10	2	-4.17

32	CID165377164	-15.5	493.59	3	0	5.97
33	CID36462 (etoposide)	-15.5	588.18	12	3	-0.14

Table S2. ADMET prediction for hit compound and its analogs

Criteria		Compounds						Unit
		CID162372098	CID162372085	CID162372077	CID156619937	CID162372088	CID36462*	
Absorption	Water solubility	-5.844	-4.896	-4.854	-4.9	-5.844	-3.487	Log (mol/L)
	Caco2 permeablity	0.67	0.402	0.316	0.428	0.67	0.403	log (Papp in 10^{-6} cm/s)
	Human intestinal absorption	100	91.432	89.511	91.424	100	75.614	%
	P-glycoprotein substrate	No	Yes	Yes	Yes	No	Yes	Yes/No
	P-glycoprotein inhibitor I	Yes	No	No	No	Yes	Yes	Yes/No
Distribution	VDss (human)	-0.795	-1.235	-1.224	-1.243	-0.795	-0.218	Log L/kg
	BBB permeability	-1.444	-1.394	-1.415	-1.385	-1.444	-1.567	Log BB
Metabolism	CYP2D6 substrate	No	No	No	No	No	No	Yes/No
	CYP2D6 inhibitor	No	No	No	No	No	No	Yes/No
	CYP3A4 substrate	Yes	Yes	Yes	Yes	Yes	Yes	Yes/No
	CYP3A4 inhibitor	No	No	No	No	No	No	Yes/No
Excretion	Total clearance	-0.302	-0.274	-0.411	-0.459	-0.302	-0.068	Log (ml/min/kg)
	Rental OCT2 substrate	No	No	No	No	No	No	Yes/No
Toxicity	AMES toxicity	No	No	Yes	No	No	No	Yes/No
	Max. Tolerated dose (human)	0.48	0.207	0.154	0.202	0.48	0.171	log (mg/kg/day)

hERG I inhibitor	No	No	No	No	No	No	Yes/No
hERG II inhibitor	Yes	No	No	No	Yes	No	Yes/No
Oral rat accute Toxicity (LD50)	2.361	0.2583	2.568	2.584	2.361	3.25	Mol/kg
Oral rat chronic Toxicity (LOAEL)	0.127	0.705	0.771	0.722	0.127	2.429	Log (mg/kg_bw/day)
Hepatotoxicity	No	No	No	No	No	No	Yes/No
Skin sensitization	No	No	No	No	No	No	Yes/No
T. pyriformis toxicity	0.285	0.285	0.285	0.285	0.285	0.285	log ug/L
Minnow toxicity	-7.947	-3.665	-3.604	-3.609	-7.947	2.217	log mM

CID36462 is etoposide*
